# Effectiveness of Proximal Interphalangeal Joint Block Orthosis and Metacarpophalangeal Joint Block Orthosis in the Management of Trigger Finger: A Prospective Observational Study

**DOI:** 10.7759/cureus.106131

**Published:** 2026-03-30

**Authors:** Mohit Kataruka, Jalil SK, Souvik Bhattacharjee, Puja Sannyashi

**Affiliations:** 1 Physical Medicine and Rehabilitation, All India Institute of Medical Sciences (AIIMS) Kalyani, Kalyani, IND

**Keywords:** mcpj block, orthosis, pipj block, splinting, trigger finger

## Abstract

Background: Orthotic intervention is a non-invasive and commonly used treatment for trigger finger (TF). Among the various splinting options, the comparative effectiveness of proximal interphalangeal joint block orthosis (PIPJ-BO) and metacarpophalangeal joint block orthosis (MCPJ-BO) remains inadequately explored. This study compared the clinical effectiveness of PIPJ-BO and MCPJ-BO in reducing pain, symptom severity, and functional limitations in patients with TF.

Methods: A prospective observational study was conducted at a tertiary care hospital. Forty-six patients diagnosed with TF (Quinnell grade ≥2) were assigned to either PIPJ-BO (Group A) or MCPJ-BO (Group B). Both groups wore the orthosis 18 hours daily for eight weeks and performed tendon gliding exercises. Outcomes assessed at baseline, four weeks, and eight weeks included the Quinnell grade, Numeric Pain Rating Scale (NPRS), grip strength, and triggering episodes. Data were analyzed using Mann-Whitney U and Wilcoxon signed-rank tests.

Results: Both groups showed significant within-group improvements in Q-grade and NPRS over time (p < .001), with greater effect sizes in Group A. However, between-group differences at all time points were not statistically significant (p > .05). Grip strength improved numerically but not significantly.

Discussion: Both PIPJ-BO and MCPJ-BO were effective in the conservative management of TF. While intergroup differences were not statistically significant, PIPJ-BO showed a slightly greater reduction in symptoms. Early orthotic intervention remains beneficial and should be tailored to patient-specific factors.

## Introduction

Trigger finger (TF), also referred to as "stenosing tenosynovitis," is a hand condition characterized by locking, clicking, or catching of a finger during flexion and extension due to impaired tendon gliding through a thickened A1 pulley [1]. This condition leads to pain, limited motion, and impaired hand function if left untreated. The etiology is often idiopathic but may involve repetitive trauma, inflammatory conditions, or metabolic diseases such as diabetes mellitus [2]. The prevalence of TF in the general adult population is estimated at 2% to 3%, with a higher incidence among women and individuals with systemic comorbidities. In diabetic patients, the prevalence ranges from 5% to 20%, likely due to glycation-related tendon changes and altered microvascularity [2,3]. The thumb and ring fingers of the dominant hand are most frequently affected [3].

Management strategies for TF range from conservative options, including orthosis, corticosteroid injection, activity modification, and surgical release of the A1 pulley. Conservative management is typically attempted first, especially in early- or moderate-grade TF. Among these, orthotic treatment is a non-invasive approach that can reduce tendon friction and inflammation, with a success rate of 40% to 93% [1,4]. Several orthosis designs have been proposed for TF management, including metacarpophalangeal joint blocking orthosis (MCPJ-BO) and proximal interphalangeal joint blocking orthosis (PIPJ-BO) [5,6]. PIPJ-BO aims to reduce differential glide between the flexor digitorum superficialis and profundus tendons, which is particularly effective in reducing symptoms associated with tendon entrapment [4,5].

Despite their clinical use, comparative data on the effectiveness of MCPJ-BO and PIPJ-BO remain limited. Therefore, this study aims to compare the clinical outcomes of these two orthotic strategies in patients with TF, focusing on symptom severity, functional improvement, and patient satisfaction. The results aim to inform orthotic prescription practices and contribute to evidence-based conservative treatment protocols for TF.

## Materials and methods

A prospective observational study was conducted at the OPD of the Department of Physical Medicine and Rehabilitation, All India Institute of Medical Sciences (AIIMS), Kalyani, from November 2024 to June 2025, including patients with TF who received either a PIPJ-BO or an MCPJ-BO after obtaining institutional ethical clearance and informed patient consent.

Patients with symptoms for more than three months, aged 18-65 years, with a diagnosis of TF for more than one month, having a Quinnell grade of 2 or more, were included in this study, whereas patients with a previous history of surgery for TF or trauma on the affected hand, history of corticosteroid injections in the affected digit in the past three months, coexisting hand or wrist disease affecting function that could interfere with intervention or assessments, rheumatoid arthritis or carpal tunnel syndrome, or hand deformity were excluded from this study.

Patients with a diagnosis of TF were assessed using the Quinnell classification and then given one of the following orthoses: PIPJ-BO or an MCPJ-BO. Both orthoses reduce the gliding and irritation of the tendon over the A1 pulley.

**Figure 1:**
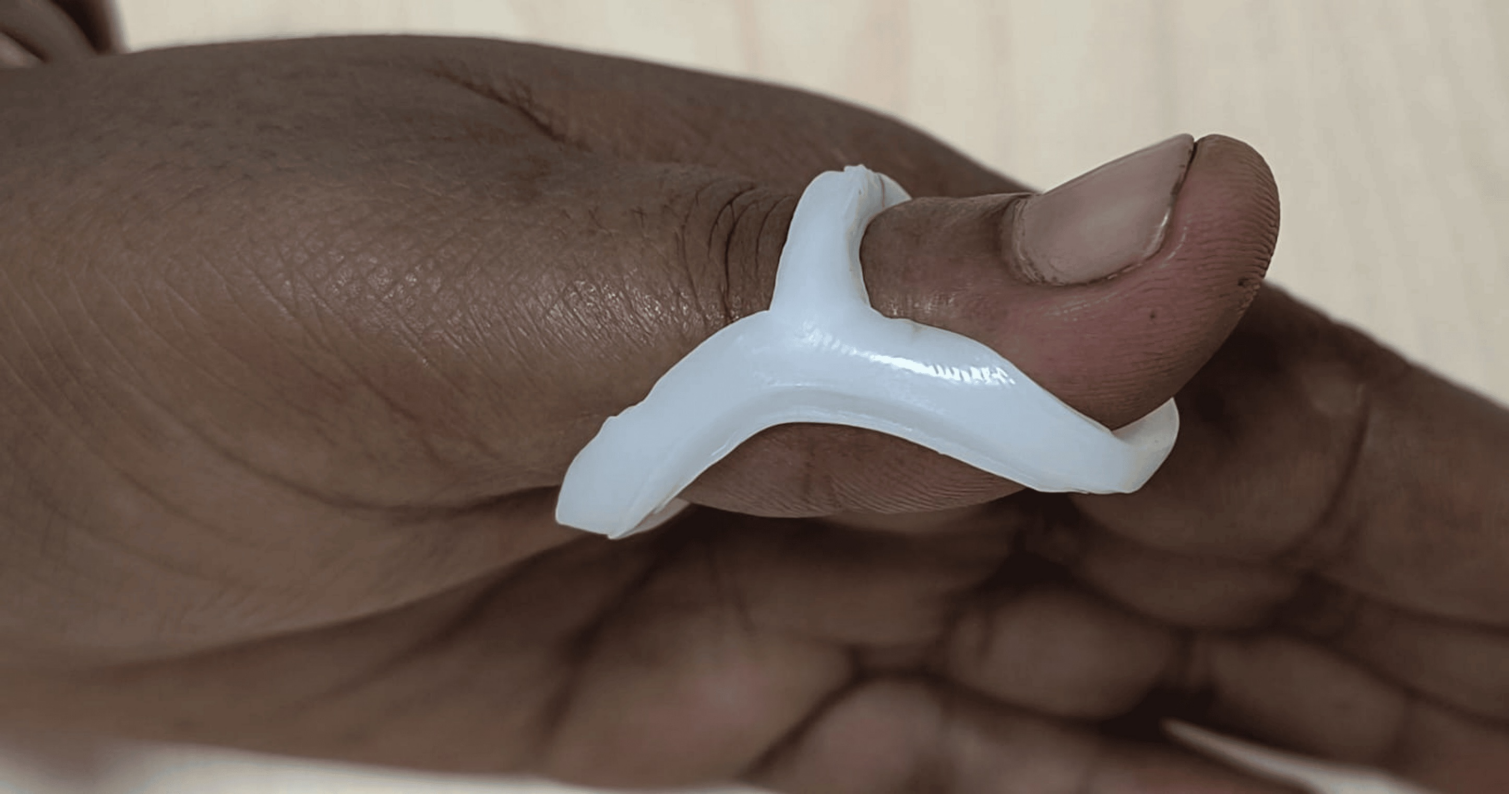
Proximal interphalangeal block orthosis (PIPJ-BO)

**Figure 2:**
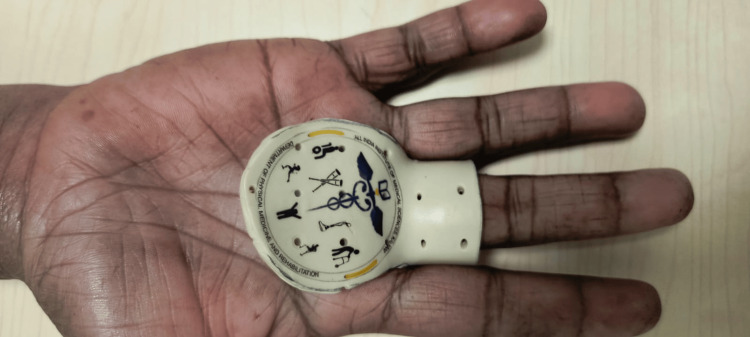
Metacarpophalangeal joint block orthosis (MCPJ-BO)

Participants were assigned to one of the following observation groups following a computer-generated randomization technique: Group A (PIPJ-BO) (Figure 1) or Group B (MCPJ-BO) (Figure 2).

Both groups were instructed to wear the orthosis for four weeks, 18 hours a day. Patients used the orthosis during the day, taking a break at night, and performed daily tendon gliding exercises, including full extension, table-top fist, hook fist, straight fist, and full fist. Each exercise lasted 20 minutes, with two to three sets per day. Exercises were taught by trained physiotherapists.

Both orthoses were fabricated from 3.2-mm low-temperature thermoplastic (LTTP) material. In MCPJ-BO, the metacarpophalangeal joint was positioned at 7-10° of flexion, whereas in PIPJ-BO, the proximal interphalangeal joint was kept straight.

Patient demographic data (such as age, gender, and underlying disease) and clinical data relating to the TF (such as onset of symptoms, affected side, affected finger, etc.) were recorded.

The outcome measures were Quinnell grading for TF for clinical severity, Numeric Pain Rating Scale (NPRS) for pain intensity, number of triggering episodes in 10 active fists, grip strength measured using a hand dynamometer (kg) [7], and Participant Perceived Improvement in Symptoms (PPIS) rating scale [8]. These outcomes were assessed at baseline, at four weeks, and at eight weeks post-intervention. In addition, any complications and patient satisfaction were also collected.

The sample size was calculated using the G*Power software version 3.1.9.7 (Heinrich Heine University Düsseldorf, Düsseldorf, Germany). Considering a two-group comparison, an effect size of 0.7, an alpha error probability of 0.05, and a power of 0.80 were used. The analysis yielded a required sample size of 52 participants, with 26 subjects per group.

Data were analyzed using IBM SPSS Statistics for Windows, Version 27 (Released 2020; IBM Corp., Armonk, New York). Descriptive data were expressed as the mean ± standard deviation or median with an interquartile range, as appropriate. The Friedman test was used to compare outcome measures within the same group, and the Mann-Whitney U test was used to compare independent groups. A p-value < 0.05 was considered statistically significant.

## Results

A total of 65 patients were included in this study, of whom 13 were excluded due to exclusion criteria. This study included 52 patients, of whom six were lost to follow-up. Each group includes 23 patients, with Group A having 18 females and five males and Group B also having 18 females and five males (Table 1).

**Table 1:** Demographic distribution of Group A vs. Group B. The categorical variables (sex, site, fingers, job) are presented as percentages. Continuous data (age) are presented as mean ± standard deviation.

Variable		Group A (n= 23)	Group B (n= 23)
Sex distribution	Female	18 (78.3%)	18 (78.3%)
	Male	5 (21.7%)	5 (21.7%)
Site affected	Left	11 (47.8%)	12 (52.2%)
	Right	12 (52.2%)	11 (47.8%)
Finger affected	Index	3 (14%)	2 (9%)
	Middle	4 (17%)	5 (22%)
	Ring	4 (17%)	6 (26%)
	Thumb	12 (52%)	10 (43%)
Job	Household	15 (65%)	14 (61%)
	Labor	5 (22%)	6 (26%)
	Others	3 (13%)	3 (13%)
Age mean in years		43±8	45±7

In Group A, there was a significant improvement in Quinnel's grading (1.08 ± 0.95), NPRS (2.65 ± 2.25), grip strength measured with the hand dynamometer (16.14 ± 7.25), PPIS (1.78 ± 1.24), and number of triggering episodes (2.26 ± 3.91) up to eight weeks (Table 2).

**Table 2:** A significant improvement was observed in Quinnell's grading, NPRS, grip strength measured with the hand dynamometer, PPIS, and the number of triggering episodes up to eight weeks in Group A. * The marked data showed a statistically significant difference. A p-value of <0.05 is considered statistically significant. D0: at the start; D1: after four weeks; D2: after eight weeks; NPRS: Numeric Pain Rating Scale; PPIS: Participant Perceived Improvement in Symptoms.

Assessment		Data in mean ± SD	Follow-up visit comparison	p-value (Friedman test)
Quinnell grading	DO	2.67 (±0.93)	D0–D1	0.008*
	D1	1.65 (± 0.83)	D1–D2	0.047*
	D2	1.08 (± 0.95)	D0–D2	0.001*
NPRS	DO	7.83 (±1.12)	D0–D1	0.002*
	D1	4.52 (±1.80)	D1–D2	0.012*
	D2	2.65 (±2.25)	D0–D2	0.001*
Grip Strength	DO	13.91 (±4.87)	D0–D1	0.010*
	D1	15.843 (±7.28)	D1–D2	0.210
	D2	16.14 (±7.25)	D0–D2	0.001*
PPIS	DO	0	D0–D1	0.039*
	D1	1.956 (±0.56)	D1–D2	0.022*
	D2	1.78 (±1.24)	D0–D2	0.001*
Catch	DO	8.44 (±3.34)	D0–D1	0.066
	D1	5.304 (±4.42)	D1–D2	0.013*
	D2	2.26 (±3.91)	D0–D2	0.001*

In Group B, there was also a significant improvement in Quinnel's grading (0.96±1.07), NPRS (3.39±2.06), grip strength measured with the hand dynamometer (13.87±5.09), PPIS (1.61±0.94), and number of triggering episodes (2.35±3.93), up to eight weeks (Table 3).

**Table 3:** A significant improvement was observed in Quinnell's grading, NPRS, grip strength measured with the hand dynamometer, PPIS, and the number of triggering episodes up to eight weeks in Group B. * The marked data showed a statistically significant difference. A p-value of <0.05 is considered statistically significant. D0: at the start; D1: after four weeks; D2: after eight weeks; NPRS: Numeric Pain Rating Scale; PPIS: Participant Perceived Improvement in Symptoms.

Assessment		Data in mean ± SD	Follow-up visit comparison	p-value (Friedman test)
Quinnell Grading	DO	2.39 (±0.78)	D0–D1	0.027*
	D1	1.652 (±0.98)	D1–D2	0.015*
	D2	0.96 (±1.07)	D0–D2	0.001*
NPRS	DO	7.347 (±1.34)	D0–D1	0.001*
	D1	4.896 (±2.28)	D1–D2	0.018*
	D2	3.39 (±2.06)	D0–D2	0.001*
Grip Strength	DO	12.28 (±3.90)	D0–D1	0.338
	D1	12.934 (±5.28)	D1–D2	0.101
	D2	13.87 (±5.09)	D0–D2	0.018*
PPIS	DO	0	D0–D1	0.554
	D1	1.91 (±0.514)	D1–D2	0.034*
	D2	1.61 (±0.94)	D0–D2	0.001*
Catch	DO	7.91 (±3.23)	D0–D1	0.104
	D1	4.65 (±4.65)	D1–D2	0.040*
	D2	2.35 (±3.93)	D0–D2	0.001*

No statistically significant differences were observed between the groups at baseline, after four weeks, or at eight weeks post-intervention in any parameter (Tables 4-8).

**Table 4:** Significant changes were observed in Quinnell grading over time (D0, D2) in both groups, but there was no statistical difference between the groups. * The marked data showed a statistically significant difference. A p-value of <0.05 is considered statistically significant. D0: at the start; D1: after four weeks; D2: after eight weeks.

Assessment of Quinnell grading	Group A (mean ± SD)	Group B (mean ± SD)	Intergroup p-value (Mann-Whitney U test)
D0	2.67 (±0.93)	2.39 (±0.78)	0.183
D1	1.65 (± 0.83)	1.652 (±0.98)	0.795
D2	1.08 (± 0.95)	0.96 (±1.07)	0.500
P-value (Friedman test)	<0.001*	<0.001*	-

**Table 5:** Significant changes were observed in the NPRS over time (D0, D2) in both groups, but there is no statistical difference between the groups. * The marked data showed a statistically significant difference. A p-value of <0.05 is considered statistically significant. D0: at the start; D1: after four weeks; D2: after eight weeks; NPRS: Numeric Pain Rating Scale.

Assessment of NPRS	Group A (mean ± SD)	Group B (mean ± SD)	Intergroup p-value (Mann-Whitney U test)
D0	7.83 (±1.12)	7.347 (±1.34)	0.191
D1	4.52 (±1.80)	4.896 (±2.28)	0.850
D2	2.65 (±2.25)	3.39 (±2.06)	0.126
P-value (Friedman test)	<0.001*	<0.001*	-

**Table 6:** Significant changes were observed in grip strength over time (D0, D2) in both groups, but there is no statistical difference between the groups. * The marked data showed a statistically significant difference. A p-value of <0.05 is considered statistically significant. D0: at the start; D1: after four weeks; D2: after eight weeks.

Assessment of grip strength (kg)	Group A (mean ± SD)	Group B (mean ± SD)	Intergroup p-value (Mann-Whitney U test)
D0	13.91 (±4.87)	12.28 (±3.90)	0.396
D1	15.843 (±7.28)	12.934 (±5.28)	0.150
D2	16.14 (±7.25)	13.87 (±5.09)	0.205
P-value (Friedman test)	<0.001*	0.018*	-

**Table 7:** Significant changes were observed in the PPIS over time (D0, D2) in both groups, but there is no statistical difference between the groups. * The marked data showed a statistically significant difference. A p-value of <0.05 is considered statistically significant. D0: at the start; D1: after four weeks; D2: after eight weeks; PPIS: Participant Perceived Improvement in Symptoms.

Assessment of PPIS	Group A (mean ± SD)	Group B (mean ± SD)	Intergroup p-value (Mann-Whitney U test)
D0	0	0	0.299
D1	1.956 (±0.56)	1.91 (±0.514)	0.527
D2	1.78 (±1.24)	1.61 (±0.94)	0.968
P-value (Friedman test)	<0.001*	<0.001*	-

**Table 8:** Significant changes were observed in the number of triggering episodes over time (D0, D2) in both groups, but there is no statistical difference between the groups. * The marked data showed a statistically significant difference. A p-value of <0.05 is considered statistically significant. D0: at the start; D1: after four weeks; D2: after eight weeks.

Assessment of catch in an active fist	Group A (mean ± SD)	Group B (mean ± SD)	Intergroup p-value (Mann-Whitney U test)
D0	8.44 (±3.34)	7.91 (±3.23)	0.299
D1	5.304 (±4.42)	4.65 (±4.65)	0.527
D2	2.26 (±3.91)	2.35 (±3.93)	0.968
P-value (Friedman test)	0.001*	0.001*	-

## Discussion

TF is stenosing tenosynovitis, characterized by locking, clicking, or catching of a finger during flexion and extension due to impaired tendon gliding through a thickened A1 pulley [3]. This condition leads to pain, limited motion, and impaired hand function if left untreated. Both the PIPJ-BO and MCPJ-BO reduce symptoms in patients with TF when worn for four weeks. This study was conducted to assess the efficacy of the two and compare them.

In our study, we included 23 patients in each group, and both groups received either PIPJ-BO or MCPJ-BO. Both groups showed significant improvement in Quinnell grade, NPRS, number of triggering episodes, and PPIS over eight weeks, and this improvement persisted even after discontinuation of the orthosis at four weeks.

Colbourn et al.'s study, the statistical comparison of pre- to post-NPRS scores, revealed a significant decrease in pain ratings among participants (t = 4.294; df = 27; p < 0.001) [8]. In our study, we found similar results using MCPJ-BO. Comparison of pre- and post-triggering event scores demonstrated a significant decrease in the number of triggering events (t = 3.808; p < 0.001). In our study, we found similar results with MCPJ-BO (p < 0.05).

Teo et al. found that both PIPJ-BO and MCPJ-BO showed significant improvement in pain and Quick DASH scores over time, but only the PIPJ-BO group demonstrated a statistically significant improvement after treatment [9]. In our study, we found significant improvements in pain, Quinnell grading, and grip strength in both groups, but there was no statistically significant difference between the groups.

Tarbhai et al. reported that subjects who wore the MCP joint-blocking splint rated their comfort higher than those who wore the DIP joint-blocking splint. The selection of the splint design may depend on the subject’s clinical presentation, occupation, and leisure activities [10]. In our study, we found significant improvement in PPIS in both groups, but no statistically significant difference was observed between them.

These findings align with previous studies supporting orthotic intervention as an effective conservative management strategy for TF [4-6]. The lack of intergroup differences may reflect comparable biomechanical efficacy in reducing tendon friction across both orthotic designs. The greater improvement observed in the PIPJ group could be due to reduced differential glide between the FDS and FDP tendons, as described in prior work [4].

It is also worth noting that patient-specific factors, such as hand dominance, digit involvement, and compliance, may influence the effectiveness of orthotics. While Group A showed more consistent within-group gains, a larger sample size and randomized design could further clarify these trends.

This study adds to existing evidence by directly comparing two widely used orthotic approaches. Unlike prior studies that have focused on steroid injections or surgical outcomes [1,11], our work supports refining orthotic prescriptions based on symptom grade and digit involvement.

Atthakomol et al. reported that splinting alone can improve pain reduction and symptom or functional improvement up to one year [12]. In our study, we have also found similar results, though we followed the patients until eight weeks.

According to Nadar [13], the orthosis resolved TF symptoms in 53.6% of participants at six weeks post-intervention, compared with the hand therapy control group, which showed no significant improvement. The short-form version of the Disabilities of the Arm, Shoulder, and Hand (DASH) disability score was significantly reduced after wearing the orthosis. We found similar results in our study.

Yendi et al. showed that the MCPJ-BO resulted in better functional status than the relative motion extension orthosis and found no significant differences between the two orthoses regarding satisfaction with the orthosis [14]. In our study, we compared PIPJ-BO and MCPJ-BO and found that both had significant effects on pain, triggering episodes, and hand grip strength.

Minkhorst et al. compared conservative treatment vs. combined therapy for TF and did not find any significant advantages of adding injection to orthosis, which implies orthosis alone has a significant role in the treatment of TF [6]. We had also found significant changes with the orthosis in our study.

McKenna et al. noted in their meta-analysis that splinting is an effective short-term conservative measure for TF and that a proximal interphalangeal block orthosis is superior in functional outcomes, patient satisfaction, and cost-effectiveness. They recommended its continuous use for at least six weeks, though we found similar results for PIPJ-BO with MCPJ-BO [15].

The main limitations of this study include a small sample size, a lack of proper randomization, and a relatively short follow-up period. Patient compliance and orthosis wear time were not formally recorded, which may introduce variability in the results. In addition, the long-term recurrence rate was not assessed. Many participants did not comply with the bracing schedule; some reported functional restrictions following orthosis use, and some orthoses also broke. A functional assessment scale was not included in this study. Additionally, there were more female patients in this study, which may skew the bias. Future studies with randomized controlled designs and long-term follow-up would be beneficial for confirming these findings and establishing clinical guidelines for orthotic management in TF.

## Conclusions

This prospective observational study supports the effectiveness of both PIPJ-BO and MCPJ-BO in reducing pain and symptom severity in patients with TF. Although intergroup differences were not statistically significant, the PIPJ-BO group demonstrated slightly greater clinical improvement. Orthotic intervention should be considered an early and viable treatment option, with individual selection based on digit involvement, occupational demands, and patient preference.
